# Socioeconomic Disparities in Foods/Beverages and Nutrients Consumed by U.S. Adolescents When Snacking: National Health and Nutrition Examination Survey 2005–2018

**DOI:** 10.3390/nu13082530

**Published:** 2021-07-24

**Authors:** Navika Gangrade, Janet Figueroa, Tashara M. Leak

**Affiliations:** 1 Division of Nutritional Sciences, Cornell University, Ithaca, NY 14853, USA; tml226@cornell.edu; 2 Department of Pediatrics, Emory University School of Medicine, Atlanta, GA 30322, USA; janet.figueroa@emory.edu

**Keywords:** adolescent, snacking, disparities, socioeconomic status, NHANES, dietary patterns

## Abstract

Snacking contributes a significant portion of adolescents’ daily energy intake and is associated with poor overall diet and increased body mass index. Adolescents from low socioeconomic status (SES) households have poorer snacking behaviors than their higher-SES counterparts. However, it is unclear if the types of food/beverages and nutrients consumed during snacking differ by SES among adolescents. Therefore, this study examines SES disparities in the aforementioned snacking characteristics by analyzing the data of 7132 adolescents (12–19 years) from the National Health and Nutrition Examination Survey 2005–2018. Results reveal that adolescents from low-income households (poverty-to-income ratio (PIR) ≤ 1.3) have lower odds of consuming the food/beverage categories “Milk and Dairy” (aOR: 0.74; 95% CI: 0.58-0.95; *p* = 0.007) and “Fruits” (aOR: 0.62, 95% CI: 0.50–0.78; *p* = 0.001) as snacks and higher odds of consuming “Beverages” (aOR: 1.45; 95% CI: 1.19-1.76; *p* = 0.001) compared to those from high-income households (PIR > 3.5). Additionally, adolescents from low- and middle-income (PIR > 1.3–3.5) households consume more added sugar (7.98 and 7.78 g vs. 6.66 g; *p* = 0.012, *p* = 0.026) and less fiber (0.78 and 0.77 g vs. 0.84 g; *p* = 0.044, *p* = 0.019) from snacks compared to their high-income counterparts. Future research is necessary to understand factors that influence snacking among adolescents, and interventions are needed, especially for adolescents from low-SES communities.

## 1. Introduction

Adolescents from low socioeconomic status (SES) households are at greater risk of obesity than their higher-SES counterparts [1,2]. One way to reduce obesity risk is to improve snacking behaviors. Snacks, often consumed between meals (e.g., breakfast, lunch, and dinner) [3], account for 22% of adolescents’ daily energy intake [4]. In addition, adolescents consume an average of 284.54 calories (kcal) per snacking occasion [5]. Moreover, there is evidence that poor snacking behaviors, such as greater kcal consumed from snacks and more frequent consumption of energy-dense, nutrient-poor snacks, are positively associated with obesity among adolescents [5,6,7]. What remains less clear is if there are SES differences in snacking behaviors, such as snacking frequency, foods/beverages consumed as snacks, and nutrients consumed from snacks.

On average, adolescents have 1.77 snacking occasions per day [5], and it remains unclear if there are differences in snacking frequency by SES. A study by Tripicchio et al. analyzed data from the National Health and Nutrition Examination Survey (NHANES) 2005–2016 and found no SES differences in snacking frequency among adolescents (12–19 years) [5]. However, the analysis only examined SES differences between adolescents from households with a poverty-to-income ratio (PIR) of greater than and less than 1.25. Additionally, analysis of data from Project EAT (Eating Among Teens), a longitudinal study in urban areas of Minnesota, USA, also found no significant differences in snacking frequency by SES [8]. However, Project EAT does not include a nationally representative sample, and snacking frequency was captured through adolescents’ self-report of the number of snacking occasions per day. Thus, conclusions from this study may have limited generalizability.

In addition to snacking frequency, there may be SES differences in the types of foods/beverages consumed as snacks and the nutrient composition of snacks. An analysis of NHANES 2005–2006 revealed that the top five foods/beverages consumed as snacks by adolescents are grains, vegetables, fruits, milk, and meat/beans [9]. The authors did not examine differences in foods/beverages consumed as snacks by SES. Regarding the nutrient composition of snacks, the analysis of NHANES 2005–2016 by Tripicchio et al. reported that adolescents from low-SES households consume significantly more added sugar calories from snacks than adolescents from higher-SES households [5]. This analysis also found no differences in sodium and saturated fat consumed from snacks by SES [5]. Findings from Project EAT reveal that adolescents from low-SES households consume significantly more sugary drinks and energy-dense, nutrient-poor snacks (captured by a food frequency questionnaire) than adolescents from higher-SES households [8]. Overall, data on the types of foods/beverages consumed as snacks by adolescents are dated, with limited examination of differences by SES. In addition, only select nutrients have been examined when exploring SES differences in the nutrient composition of snacks.

The overall aim of this paper is to analyze data from a nationally representative sample of U.S. adolescents (12–19 years) participating in NHANES 2005–2018 to examine what foods/beverages and nutrients are consumed when snacking and what differences exist by SES. Findings can help guide future interventions that aim to improve snacking behaviors among adolescents from low-SES households.

## 2. Materials and Methods

### 2.1. Study Design and Population

Data from adolescents (12–19 years) who participated in NHANES 2005–2018 were included in this analysis. NHANES is an ongoing, cross-sectional study conducted by the Centers for Disease Control and Prevention (CDC) that collects nutrition and health data from a nationally representative sample of U.S. civilian, noninstitutionalized populations [10]. NHANES is conducted in two-year cycles, using a complex, multistage probability sampling method to recruit participants. Seven NHANES cycles from 2005–2018 (i.e., 2005–2006, 2007–2008, etc.) were included in this analysis. Data collection for NHANES starts with an at-home interview where sociodemographic and basic health information are collected by a trained interviewer. Adolescents 12–15 years answer questions with the assistance of an adult, and adolescents 16–19 years answer questions independently.

Next, adolescents visit the NHANES Mobile Examination Center (MEC), where health measures (e.g., anthropometrics) and a 24-h dietary recall are collected. Adolescents (≥12 years) independently self-report dietary intake. A second 24 h dietary recall is collected via telephone within 3 to 10 days of the MEC visit but was not used in this present study. In this present study, only 1 day of dietary data was able to be used, since the most common types of foods/beverages consumed as snacks were described by the proportion of adolescents consuming the food/beverage category as a snack on a given day. Thus, the interviewer-administered 24 h dietary recall in the MEC was utilized, as it is more standardized and valid than the second 24 h dietary recall collected via telephone [11].

Given the research question, inclusion criteria were adolescents (12–19 years), reliable interviewer-administered 24 h dietary recall in the MEC, consumption of at least one snack item other than plain/tap water, and complete household poverty-to-income ratio (PIR) data (2948 excluded). The final analytic sample included 7132 adolescents. 

Adolescents 12–17 years provided assent with parental consent, and adolescents 18–19 years provided consent. NHANES was approved by the National Center for Health Statistics Research Ethics Review Board (protocol #2005-6 for years 2007–2010, #2011-17 for years 2011–2016, and #2011-17 and #2018-01 for years 2017–2018) [12]. NHANES data are publicly available and de-identified, and university Institutional Review Board approval was not necessary. 

### 2.2. Measures

#### 2.2.1. Sociodemographic Characteristics

Sex (male or female) and race/ethnicity (non-Hispanic White, non-Hispanic Black, Mexican American, Other non-Hispanic, and Other Hispanic) were self-reported. Household PIR was utilized to operationalize SES, and it is calculated by dividing family income by the poverty threshold specific to the survey year and accounting for family size. PIR was characterized into three categories: low-income (PIR ≤ 1.3), middle-income (PIR > 1.3–3.5), and high-income (PIR > 3.5) [13]. As an estimation, a PIR of 1.3 equates to an annual income of $34,444 for a family of four, and a PIR of 3.5 equates to $92,733 for a family of four [14].

#### 2.2.2. Dietary Intake 

Dietary intake was obtained through the first interviewer-administered 24 h dietary recall in the MEC. The United States Department of Agriculture (USDA) Automated Multiple-Pass Method was used to collect the dietary recalls, and the following information about all foods/beverages consumed was collected: name of eating occasion, time eaten, where obtained, eaten at home or not, description of the food/beverage, additions to the food/beverage, and amount of food/beverage consumed [15]. The selected “name of eating occasion” was used to identify snack items (i.e., snack, drink, extended consumption, merienda, entre comida, botana, bocadillo, tentempie, or bebida). The term “snack item” will be used hereafter to refer to each separate snack food/beverage reported by the participant.

In the dataset, each snack item was given a Food and Nutrient Database for Dietary Studies (FNDDS) code, and the Food Patterns Equivalent Database (FPED) was used to convert FNDDS codes into USDA What We Eat in America (WWEIA) Food/Beverage Categories. In this study, we will report on the top five WWEIA Food/Beverage Categories by the proportion of adolescents consuming it as a snack item. In addition, we will report the average number of snack items consumed, average amount of energy per snack item, proportion of total energy intake from snack items, and mean nutrient densities of snack items (grams, milligrams, or micrograms of the nutrient per 100 kcal of the snack item). The nutrients examined in this study were the three macronutrients and dietary components of public health interest according to the USDA Dietary Guidelines for Americans (i.e., saturated fat, added sugar, fiber, sodium) [16].

### 2.3. Analyses

Survey weights, strata, and clustering variables were used to account for the complex, multistage probability sampling design used in NHANES [17,18]. A combined (seven-survey cycle) weight was calculated using the dietary day 1 sample weights in accordance with NHANES methods [19]. Categorical variables were summarized with weighted proportions, and continuous variables were described using weighted means ± standard error (SE). Rao–Scott Chi-Square tests were used for examining associations between categorical data. Survey-weighted logistic regression models were used to estimate adjusted odds ratios (aOR) with 95% confidence intervals. Survey-weighted multiple linear regression models were run to estimate adjusted least squares (LS) means for comparisons of continuous data. The normality of the fitted residuals from the linear models was assessed using histograms and Q–Q plots. If distributions were highly skewed, *p*-values were reported using square-root transformed nutrient values. All models controlled for sex, age (years), and race/ethnicity to produce adjusted estimates. The significance level was set at *p* < 0.05, and *p*-values were also adjusted for multiple pairwise comparisons using the Tukey–Kramer adjustment. All analyses were conducted using SAS Version 9.4 (Cary, NC, USA).

## 3. Results

Weighted proportions for individual and household characteristics of the study sample (*n* = 7132) are presented in Table 1. Approximately half (51.8%) of the participants were 12–15 years, and half (48.2%) were 16–19 years. Most participants were non-Hispanic White (29.7%), and 26.3% were non-Hispanic Black, 23.9% were Mexican American, 11.3% were Other non-Hispanic, and 8.8% were Other Hispanic. About 32.5% of the adolescents resided in a low-income household (PIR ≤ 1.3), 36.0% in a middle-income household (PIR > 1.3–3.5), and 31.5% in a high-income household (PIR > 3.5).

On average, adolescents consumed 2.94 snack items per day. There were no significant differences in the number of snack items consumed by PIR, with those from low-, middle-, and high-income households consuming 2.90, 2.92, and 2.99 snack items per day, respectively. Per snack item, adolescents consumed an average of 210.89 kcal and 10.27% of their total energy intake. Those from low-income households consumed significantly more calories per snack item than those from high-income households (220.25 kcal vs. 194.94 kcal; *p* = 0.023). Additionally, adolescents from low- and middle-income households had a higher percentage of calories from snacks than those from high-income households (10.85% and 10.54% vs. 9.44%; *p* < 0.001 and *p* = 0.006). 

### 3.1. Types of Foods and Beverages Consumed as Snack Items and Differences by SES

Table 2 describes the five most common WWEIA Food/Beverage Categories consumed as a snack item. The “Snacks and Sweets” category (e.g., savory snacks, crackers, candy) was consumed as a snack item by 73.3% of adolescents. “Beverages” (e.g., sweetened beverages, 100% juice, diet beverages, and coffee and tea) were consumed by 51.9% of adolescents. “Milk and Dairy” was the third most consumed food/beverage category, with a quarter (25.0%) of adolescents consuming it as a snack item. “Fruits” were consumed as a snack item by 21.0% of adolescents. Lastly, “Grains”, which include quick breads and bakery products and ready-to-eat cereals, were consumed by 15.4% of adolescents. In terms of overall diet, snacking contributed to approximately 68.7% of adolescents’ daily “Snacks and Sweets” intake, 40.2% of their “Beverages” intake, 22.2% of their “Milk and Dairy” intake, 49.4% of their “Fruits” intake, and 11.1% of their “Grains” intake.

There were also differences in the odds of consuming a particular food/beverage category as a snack item by income (Table 3). Adolescents from low-income households had approximately 45% higher odds of consuming “Beverages” as a snack item than those from high-income households (aOR: 1.45; 95% CI: 1.19–1.76; *p* = 0.001). However, odds of consuming “Snacks and Sweets” (aOR: 0.75; 95% CI: 0.59–0.95; *p* = 0.035), “Milk and Dairy” (aOR: 0.74; 95% CI: 0.58–0.95; *p* = 0.007), and “Fruits” (aOR: 0.62, 95% CI: 0.50–0.78; *p* = 0.001) were lower than adolescents from high-income households. There were no significant differences in odds of consuming “Grains” by SES.

### 3.2. Nutrient Composition of Snack Items and Differences by SES

Per 100 kcal of the snack item, adolescents consumed an average of 2.85 g total fat, 1.00 g saturated fat, 17.55 g carbohydrates, 0.80 g fiber, 10.68 g total sugar, 7.46 g added sugar, 103.80 mg sodium, and 1.96 g protein. In terms of overall diet, snacking contributed to approximately 25.0% of adolescents’ daily total fat intake, 25.4% of saturated fat intake, 31.6% of carbohydrate intake, 25.5% of fiber intake, 39.5% of total sugar intake, 41.9% of added sugar intake, 17.8% of sodium intake, and 16.9% of protein intake.

There were differences in nutrient intake by income (Table 4). Adolescents from low- and middle-income households consumed 2.79 and 2.81 g of total fat per 100 kcal of the snack item, respectively, while adolescents from high-income households consumed 2.96 g of total fat (*p* = 0.007 and *p* = 0.041). Similarly, those from low- and middle-income households consumed less saturated fat than those from high-income households (0.95 and 0.98 g vs. 1.07 g; *p* = 0.001 and *p* = 0.039). 

For carbohydrates, adolescents from low-income households consumed 17.78 g of the nutrient per 100 kcal of the snack item, while adolescents from high-income households consumed significantly less at 17.20 g (*p* = 0.030). Adolescents from low- and middle-income households had significantly less fiber from snack items than those from high-income households (0.78 and 0.77 g vs. 0.84 g; *p* = 0.044 and *p* = 0.019). In terms of total sugar, those from low- and middle-income households consumed 10.88 and 10.94 g per 100 kcal of the snack item, respectively, which was significantly higher than adolescents from high-income households (10.21 g; *p* = 0.037 and *p* = 0.022). For added sugar, those from low- and middle-income households also consumed significantly more (7.98 and 7.78 g) than those from high-income households (6.66 g; *p* = 0.012 and *p* = 0.026). There were no significant differences in sodium from snack items by SES. Lastly, adolescents from low- and middle-income households consumed less protein at 1.85 and 1.94 g per 100 kcal of the snack item, respectively, compared to those from high-income households, who consumed 2.08 g protein (*p* < 0.001 and *p* = 0.01).

## 4. Discussion

This study fills a critical gap in understanding SES differences in foods/beverages and nutrients consumed during snacking among a nationally representative sample of U.S. adolescents. Findings from the current study revealed that adolescents from lower-income households consume significantly more calories, “Beverages”, carbohydrates, total sugar, added sugar and less “Snacks and Sweets”, “Milk and Dairy”, “Fruits”, total fat, saturated fat, fiber, and protein from snacks than adolescents from high-income households. Of particular concern are “Beverages”, “Fruits”, and “Milk and Dairy”, as well as added sugar, fiber, and saturated fat, which are dietary components of public health interest, according to the Dietary Guidelines for Americans [16]. 

In this study, adolescents from lower-income backgrounds had higher added sugar intake from snacks than those from high-income backgrounds. This is similar to a study that utilized NHANES 2005–2016 data and found that adolescents from low-income households consumed significantly more added sugar calories from snacks than those from higher-income households [5]. However, a study that examined SES differences in added sugar intake in adolescents’ overall diet did not find differences by PIR [20]. As such, this information collectively suggests that adolescents from low-SES households consume snacks high in added sugar, such as sugar-sweetened beverages (SSBs) [8]. Disparities in SSB consumption may explain some of the difference in added sugar intake from snacks. In fact, data from this current study reveal that the odds of those from low-income households consuming “Beverages” (which includes SSBs) are higher than those from high-income households. Other studies describe similar income disparities in SSB consumption [21]. Thus, focusing on reducing SSBs consumed as snacks among adolescents from low-income households may decrease disparities in added sugar intake from snacks. 

Findings also revealed that adolescents from low-SES backgrounds consumed less fiber from snacks than their peers from high-SES backgrounds, which aligns with other studies that have found SES disparities in intake of fiber-rich foods, such as whole grains and fruits [22,23]. An analysis of NHANES 2005–2012 found that whole grain intake was higher among adolescents from high-income households compared to those from low-income households [22]. In addition, fruits are one of the main sources of dietary fiber intake among U.S. children [24], and this study reveals that those from low-income households have lower odds of consuming “Fruits” as a snack, compared to those from higher-income households. Thus, increasing intake of whole grains and fruits as snacks may improve fiber intake among adolescents from lower-income households. 

Lastly, we found that saturated fat intake from snacks was lower among adolescents from lower-income households than the high-income group. This finding may be related to the significantly lower odds of consuming “Milk and Dairy” also reported in this present study. Evidence from NHANES suggests that milk is one of the top sources of solid fat (which includes saturated fat) among U.S. children (2–18 years) [25]. As such, the lower consumption of “Milk and Dairy” among adolescents from lower-income households in this study could contribute to findings related to saturated fat. Other studies corroborate these findings, revealing that milk and dairy intake among adolescents is positively correlated with SES [26] and that milk and dairy intake is low among adolescents from low-SES backgrounds due to factors like preferences, lactose intolerance, and health beliefs among this demographic [27,28]. 

In response to the contrary findings surrounding “Snacks and Sweets”, which suggest that those from low-income households have lower odds of consuming “Snacks and Sweets” compared to those from high-income households, we explored several explanations. One explanation is that adolescents from lower-income households may be eligible for USDA food assistance programs like the Child and Adult Care Food Program (CACFP), which provides free snacks to youth that attend centers that serve a large number of children from low-income households [29]. There are food group/nutrient requirements for the snacks provided by CACFP such that they cannot include many of the foods in the “Snacks and Sweets” category [30], possibly leading to lower odds of consuming them among adolescents from lower-income backgrounds. Another explanation may be that adolescents from high-income households have greater discretionary funds [31], which they may use to purchase foods in the “Snacks and Sweets” category. Nevertheless, nutrients that are consumed from “Snacks and Sweets”, such as kcal, added sugar, and sodium, were significantly higher in lower-income groups. As such, more research may be needed to understand the findings surrounding “Snacks and Sweets”.

Ultimately, this study highlights improvements that can be made to adolescent snacking behaviors, and one area to initiate improvements is in corner stores, where adolescents from lower-income households frequently purchase snacks [32]. A study based in low-income areas of Philadelphia found that adolescents purchased an average of 2.3 food/beverage items during each corner store visit [33]. Data also reveal that adolescents’ corner store purchases are energy-dense, nutrient-poor foods/beverages [33,34]. In the aforementioned study in Philadelphia, the top five categories of items purchased from the corner store were beverages (the top two beverages were regular soda and fruit-flavored drinks [<100% fruit juice]), chips, pastry, candy, and prepared food items (e.g., sandwiches and bagels) [33]. In addition, researchers found each adolescent’s purchasing occasion contained, on average, 650.2 kcal, 25.0 g fat, 13.9 g protein, 95.5 g carbohydrates, 61.9 g sugars, 2.3 g fiber, and 786.3 mg sodium [33] As such, this information alongside results from this study highlight an opportunity to improve adolescent corner store snacking.

There are several ways to improve adolescent corner store snacking. One method is to increase the availability and accessibility of healthier snack options. A study that surveyed 403 adolescents found that they would be receptive to buying a fiber-rich product called a whole-grain snack pack, which is a whole-grain snack (e.g., granola bar, crackers) paired with a fruit or vegetable and a condiment (e.g., yogurt, dressing), if they were available at corner stores [35]. A related study found that corner store owners would also be receptive to stocking a whole-grain snack pack [36]. Making a product like a whole-grain snack pack available and accessible to adolescents may improve fiber and fruit intake from snacks. Another intervention based in Philadelphia centered on providing resources (e.g., corner store owner trainings, signage, refrigeration) to increase the availability and access of healthy foods and beverages at 192 corner stores [34]. However, one year after the intervention, they found no differences in the energy or nutrient content of corner stores purchases [34]. As such, other options to improve adolescent corner store snacking include nutrition education. An example of this is the Taster’s Choice (TC) curriculum, which is a 6th-grade health class curriculum focused on empowerment and encouraging peer promotion of healthy snack and SSB consumption from corner stores [37]. Focus group findings revealed that adolescents reduced SSB consumption after participation. Overall, strategies to address availability, accessibility, and education surrounding healthy snacks at corner stores are imperative in remediating SES snack disparities highlighted in this study. 

Though this study provides critical, translational information, it is not without limitations. For example, we only used a single 24-h recall, and there is a possibility it may not reflect usual intake, though guidelines report that a single 24-h recall is valid for estimating the average intake of a large group [11,38]. Additionally, the dietary data were self-reported, so there may be missing and/or inaccurate data due to recall and/or social desirability bias. An example of this includes misreporting of portion sizes of foods and beverages, which may result in under- or overestimations of energy intake. However, in NHANES, the computer-assisted Automated Multiple-Pass Method for obtaining dietary data is used, and research reports that this method reduces bias in the collection of energy intakes compared to other 24 h recall methods [39]. In addition, we did not include physical activity as a covariate as the questions capturing physical activity were redefined in 2011 and not comparable across multiple survey years. Lastly, NHANES is a cross-sectional study, and thus casual inferences cannot be made. Therefore, there is a need for further research on factors that influence snacking among adolescents. 

Despite these limitations, this study is the most detailed and updated description of snacking behaviors among a nationally representative sample of U.S. adolescents. Examining the types of foods/beverages and nutrients consumed while snacking by SES may help explain disparities in overall diet and obesity among adolescents, since most studies of adolescent snacking behaviors only examine snacking frequency and energy from snacks. This previously missing information is imperative in creating effective interventions and policies to improve snacking behaviors among adolescents, especially those from low-SES households. 

## 5. Conclusions

In summary, intervening on the types of foods/beverages consumed as snacks may be one way to address the childhood obesity epidemic and reduce SES disparities. Our findings provide insight into specific foods/beverages and nutrients that adolescents consume while snacking, which can be used to develop targeted intervention efforts. 

This study also provides a base for future research on snacking, including exploring snacking in relation to overall diet and examining differences between those who snack and those who do not. Furthermore, implications from this study include the need for strategies that address factors that influence snack choices at the individual, environmental, and policy levels. As such, research is also needed to understand factors that influence snacking behaviors and develop ways to improve the availability, accessibility, and affordability of healthy snacks in low-income communities. 

## Figures and Tables

**Table 1:** Demographic characteristics of U.S. adolescents (12–19 years), *n* = 7132 (NHANES 2005–2018).

			Poverty-to-Income Ratio Groups			
Characteristic	Overall		PIR ≤ 1.3 Low-Income (*n* = 2975)	PIR > 1.3–3.5 Middle-Income (*n* = 2580)	PIR > 3.5 High-Income (*n* = 1577)	*p*-Value ^b^
	*n*	%	% (SE)	% (SE)	% (SE)	
Total ^a^	7132	100	32.5 (1.52)	36.0 (1.24)	31.5 (1.43)	
Sex						0.11
Male	3620	50.8	48.1 (1.28)	52.8 (1.57)	51.2 (1.86)	
Female	3512	49.2	51.9 (1.28)	47.2 (1.57)	48.8 (1.86)	
Age						**0.031**
12–15	3693	51.8	47.2 (1.74)	53.2 (1.54)	52.7 (1.78)	
16–19	3439	48.2	52.8 (1.74)	46.8 (1.54)	47.3 (1.78)	
Race/ethnicity						**<0.001**
Non-Hispanic White	2121	29.7	41.3 (2.57)	58.1 (2.47)	77.4 (1.52)	
Non-Hispanic Black	1878	26.3	20.7 (1.93)	13.5 (1.22)	7.0 (0.78)	
Mexican American	1701	23.9	21.3 (1.96)	12.9 (1.17)	5.1 (0.72)	
Other Non-Hispanic	807	11.3	7.5 (0.96)	8.7 (1.00)	7.9 (0.95)	
Other Hispanic	625	8.8	9.2 (1.17)	6.8 (0.91)	2.6 (0.42)	

^a^ Row % displayed for “Total”. All other percentages are column %. ^b^ Comparisons of demographic variables across PIR groups were conducted using Rao–Scott Chi-Square tests. *p* < 0.05 are shown in bold.

**Table 2:** Proportion of adolescents consuming What We Eat in America food/beverage categories as a snack item on a given day by poverty-to-income ratio (PIR) from NHANES 2005–2018 (*n* = 7132).

		Poverty-to-Income Ratio Groups			
Top 5 Food/Beverage Categories	All Adolescents (*n* = 7132)	PIR ≤ 1.3 Low-Income (*n* = 2975)	PIR > 1.3–3.5 Middle-Income (*n* = 2580)	PIR > 3.5 High-Income (*n* = 1577)	*p*-Value ^a^
	% (SE)	% (SE)	% (SE)	% (SE)	
Snacks and Sweets	73.3 (0.90)	71.2 (1.20)	72.4 (1.53)	76.4 (1.75)	**0.038**
Beverages	51.9 (0.93)	55.1 (1.55)	52.8 (1.33)	47.7 (1.82)	**0.004**
Milk and Dairy	25.0 (0.88)	21.4 (1.05)	25.9 (1.53)	27.8 (1.95)	**0.017**
Fruits	21.0 (0.87)	18.7 (1.10)	19.8 (1.30)	24.8 (1.73)	**0.004**
Grains	15.4 (0.82)	15.7 (1.01)	15.9 (1.25)	14.6 (1.31)	0.65

Weighted column % displayed. ^a^ Comparisons of reported food/beverage category consumption across PIR categories were conducted using Rao–Scott Chi-Square tests. *p* < 0.05 are shown in bold.

**Table 3:** Odds of adolescents consuming a What We Eat in America food/beverage category as a snack item on a given day by poverty-to-income ratio (PIR) from NHANES 2005–2018 (*n* = 7132).

Top 5 Food/Beverage Categories	Poverty-to Income Ratio	Odds Ratio	95% Confidence Interval	*p*-Value
Snacks and Sweets	PIR ≤ 1.3 low-income	0.75	0.59–0.95	**0.035**
	PIR > 1.3–3.5middle-income	0.79	0.61–1.03	0.35
Beverages	PIR ≤ 1.3 low-income	1.45	1.19–1.76	**0.001**
	PIR > 1.3–3.5 middle-income	1.28	1.05–1.57	0.44
Milk and Dairy	PIR ≤ 1.3 low-income	0.74	0.58–0.95	**0.007**
	PIR > 1.3–3.5 middle-income	0.92	0.71–1.19	0.56
Fruits	PIR ≤ 1.3 low-income	0.62	0.50–0.78	**0.001**
	PIR > 1.3–3.5 middle-income	0.69	0.54–0.89	0.20
Grains	PIR ≤ 1.3 low-income	1.03	0.81–1.31	0.98
	PIR > 1.3–3.5 middle-income	1.06	0.83–1.36	0.66

Survey-weighted logistic regression models were adjusted for sex, age (years), and race/ethnicity; adjusted odds ratios with 95% CI are shown. Referent group PIR > 3.5. *p* < 0.05 is shown in bold.

**Table 4:** Energy and nutrient consumption from snack items by poverty-to-income ratio (PIR) among adolescents from NHANES 2005–2018 (*n* = 7132).

		Poverty-to-Income Ratio		
Nutrients	All Adolescents (*n* = 7132)	PIR ≤ 1.3 Low-Income (*n* = 2975)	PIR > 1.3–3.5 Middle-Income (*n* = 2580)	PIR > 3.5 High-Income (*n* = 1577)
	LS Mean ± SE	LS Mean ± SE	LS Mean ± SE	LS Mean ± SE
Energy, kcal	210.89 ± 4.02	220.25 ± 5.90 ^a^	218.27 ± 8.66	194.94 ± 5.33
Energy, %	10.27 ± 0.15	10.85 ± 0.28 ^c^	10.54 ± 0.23 ^b^	9.44 ± 0.24
Total Fat, g	2.85 ± 0.04	2.79 ± 0.05 ^b^	2.81 ± 0.06 ^a^	2.96 ± 0.06
Saturated Fat, g	1.00 ± 0.02	0.95 ± 0.02 ^c^	0.98 ± 0.03 ^a^	1.07 ± 0.03
Carbohydrate, g	17.55 ± 0.11	17.78 ± 0.14 ^a^	17.68 ± 0.19	17.20 ± 0.19
Fiber, g	0.80 ± 0.02	0.78 ± 0.03 ^a^	0.77 ± 0.03 ^a^	0.84 ± 0.03
Total Sugar, g	10.68 ± 0.11	10.88 ± 0.19 ^a^	10.94 ± 0.18 ^a^	10.21 ± 0.19
Added Sugar, g	7.46 ± 0.14	7.98 ± 0.23 ^a^	7.78 ± 0.18 ^a^	6.66 ± 0.21
Sodium, mg	103.80 ± 3.26	99.02 ± 3.05	101.39 ± 4.37	111.02 ± 7.34
Protein, g	1.96 ± 0.04	1.85 ± 0.06 ^c^	1.94 ± 0.07 ^b^	2.08 ± 0.05

^a^ *p* < 0.05 when compared with PIR > 3.5. ^b^ *p* < 0.01 when compared with PIR > 3.5. ^c^ *p* < 0.001 when compared with PIR > 3.5. “Energy, kcal” is the average calorie amount of a snack item. “Energy, %” is the average % energy of a snack item out of a person’s total energy intake. All other nutrients are presented per 100 kcal of a snack item. Survey-weighted linear regression models were adjusted for sex, age (years), and race/ethnicity; adjusted least squares (LS)-means with standard error (SE) are shown. *p*-values for all pairwise differences across all three PIR categories were adjusted using Tukey–Kramer method.

## Data Availability

All data presented in the manuscript are publicly available through the Centers for Disease Control and Prevention site at https://wwwn.cdc.gov/nchs/nhanes/default.aspx (accessed on 27 May 2021) [40]. Additionally, SAS software was used. Operating system: Platform independent, requirements: SAS or higher, license: individual, any restrictions to use by non-academics: license needed.

## Abbreviations

CACFP: Child and Adult Care Food Program; CDC: Centers for Disease Control and Prevention; FNDDS: Food and Nutrient Database for Dietary Studies; FPED: Food Patterns Equivalent Database; kcal: calories; LS: least squares; MEC: mobile examination center; NHANES: National Health and Nutrition Examination Survey; aOR: Adjusted Odds Ratio; PIR: poverty-to-income ratio; Project EAT: Project Eating Among Teens; SE: standard error; SES: socioeconomic status; SSB: sugar-sweetened beverage; USDA: United States Department of Agriculture; WWEIA: What We Eat in America.

## References

[1] Singh G.K., Kogan M.D., van Dyck P.C., Siahpush M. (2008). Racial/Ethnic, Socioeconomic, and Behavioral Determinants of Childhood and Adolescent Obesity in the United States: Analyzing Independent and Joint Associations. Ann. Epidemiol..

[2] Frederick C.B., Snellman K., Putnam R.D. (2014). Increasing socioeconomic disparities in adolescent obesity. Proc. Natl. Acad. Sci. USA.

[3] Johnson G.H., Anderson G.H. (2010). Snacking definitions: Impact on interpretation of the literature and dietary recommendations. Crit. Rev. Food Sci. Nutr..

[4] WWEIA Data Tables: USDA ARS. https://www-ars-usda-gov.proxy.library.cornell.edu/northeast-area/beltsville-md-bhnrc/beltsville-human-nutrition-research-center/food-surveys-research-group/docs/wweia-data-tables/.

[5] Tripicchio G.L., Kachurak A., Davey A., Bailey R.L., Dabritz L.J., Fisher J.O. (2019). Associations between Snacking and Weight Status among Adolescents 12–19 Years in the United States. Nutrients.

[6] Murakami K., Livingstone M.B.E. (2016). Associations between meal and snack frequency and overweight and abdominal obesity in US children and adolescents from National Health and Nutrition Examination Survey (NHANES) 2003–2012. Br. J. Nutr..

[7] Larson N.I., Miller J.M., Watts A.W., Story M.T., Neumark-Sztainer D.R. (2016). Adolescent Snacking Behaviors Are Associated with Dietary Intake and Weight Status. J. Nutr..

[8] Larson N., Story M., Eisenberg M.E., Neumark-Sztainer D. (2016). Secular Trends in Meal and Snack Patterns among Adolescents from 1999 to 2010. J. Acad. Nutr. Diet..

[9] Sebastian R.S., Goldman J.D., Enns C.W. (2010). Snacking patterns of US adolescents: What we eat in America, NHANES 2005–2006. Food Surv. Res. Gr. Diet. Data Br..

[10] National Center for Health Statistics and Centers for Disease Control and Prevention (2017). National Health and Nutrition Examination Survey. https://www.cdc.gov/nchs/nhanes/about_nhanes.htm.

[11] Ahluwalia N., Dwyer J., Terry A., Moshfegh A., Johnson C. (2016). Update on NHANES dietary data: Focus on collection, release, analytical considerations, and uses to inform public policy. Adv. Nutr..

[12] NHANES-NCHS Research Ethics Review Board Approval. https://www-cdc-gov.proxy.library.cornell.edu/nchs/nhanes/irba98.htm.

[13] Ogden C.L., Carroll M.D., Fakhouri T.H., Hales C.M., Fryar C.D., Li X., Freedman D.S. (2018). Prevalence of obesity among youths by household income and education level of head of household-United States 2011–2014. Morb. Mortal. Wkly. Rep..

[14] How the Census Bureau Measures Poverty. https://www.census.gov/topics/income-poverty/poverty/guidance/poverty-measures.html.

[15] AMPM-Information Collected: USDA ARS. https://www-ars-usda-gov.proxy.library.cornell.edu/northeast-area/beltsville-md-bhnrc/beltsville-human-nutrition-research-center/food-surveys-research-group/docs/ampm-information-collected/.

[16] U.S. Department of Agriculture and U.S. Department of Health and Human Services (2020). Dietary Guidelines for Americans, 2020–2025.

[17] Johnson C.L., Paulose-Ram R., Ogden C.L. (2013). National Health and Nutrition Examination Survey: Analytic Guidelines, 1999–2010. Vital Health Stat. 2.

[18] Centers for Disease Control and Prevention and National Center for Health Statistics (2018). National Health and Nutrition Examination Survey: Analytic Guidelines, 2011–2014 and 2015–2016. https://wwwn.cdc.gov/nchs/nhanes/analyticguidelines.aspx.

[19] NHANES Tutorials-Module 3-Weighting. https://wwwn-cdc-gov.proxy.library.cornell.edu/nchs/nhanes/tutorials/module3.aspx.

[20] Welsh J.A., Sharma A., Cunningham S.A., Vos M.B. (2011). Consumption of added sugars and indicators of cardiovascular disease risk among US adolescents. Circulation.

[21] Han E., Powell L.M. (2013). Consumption Patterns of Sugar-Sweetened Beverages in the United States. J. Acad. Nutr. Diet..

[22] Tester J.M., Leung C.W., Leak T.M., Laraia B.A. (2017). Recent uptrend in whole-grain intake is absent for low-income adolescents, national health and nutrition examination survey, 2005–2012. Prev. Chronic Dis..

[23] Larson N.I. (2021). Nutritional problems in childhood and adolescence: A narrative review of identified disparities. Nutr. Res. Rev..

[24] Mcgill C.R., Iii V.L.F., Devareddy L. (2001). Ten-Year Trends in Fiber and Whole Grain Intakes and Food Sources for the United States Population: National Health and Nutrition Examination Survey 2001–2010. Nutrients.

[25] Slining M.M., Popkin B.M. (2013). Trends in intakes and sources of solid fats and added sugars among U.S. children and adolescents: 1994–2010. Pediatr. Obes..

[26] Neumark-Sztainer D., Story M., Dixon L.B., Resnick M.D., Blum R.W. (1997). Correlates of inadequate consumption of dairy products among adolescents. J. Nutr. Educ. Behav..

[27] Lee S., Reicks M. (2003). Environmental and behavioral factors are associated with the calcium intake of low-income adolescent girls. J. Am. Diet. Assoc..

[28] Vue H., Reicks M. (2007). Individual and Environmental Influences on Intake of Calcium-rich Food and Beverages by Young Hmong Adolescent Girls. J. Nutr. Educ. Behav..

[29] Child and Adult Care Food Program|USDA-FNS. https://www.fns.usda.gov/cacfp.

[30] Nutrition Standards for CACFP Meals and Snacks|USDA-FNS. https://www.fns.usda.gov/cacfp/meals-and-snacks.

[31] Soteriades E.S., DiFranza J.R. (2003). Parent’s Socioeconomic Status, Adolescents’ Disposable Income, and Adolescents’ Smoking Status in Massachusetts. Am. J. Public Health.

[32] Borradaile K.E., Sherman S., Veur S.S.V., McCoy T., Sandoval B., Nachmani J., Karpyn A., Foster G.D. (2009). Snacking in children: The role of urban corner stores. Pediatrics.

[33] Lent M.R., Veur S.V., Mallya G., McCoy T.A., Sanders T.A., Colby L., Tewksbury C.R., Lawman H.G., Sandoval B., Sherman S. (2014). Corner store purchases made by adults, adolescents and children: Items, nutritional characteristics and amount spent. Public Health Nutr..

[34] Lawman H.G., Veur S.V., Mallya G., McCoy T.A., Wojtanowski A., Colby L., Sanders T.A., Lent M.R., Sandoval B.A., Sherman S. (2015). Changes in quantity, spending, and nutritional characteristics of adult, adolescent and child urban corner store purchases after an environmental intervention. Prev. Med..

[35] Leak T.M., Setiono F., Gangrade N., Mudrak E. (2019). Youth willingness to purchase whole grain snack packs from New York city corner stores participating in a healthy retail program. Int. J. Environ. Res. Public Health.

[36] Leak T.M., Gangrade N., Setiono F.J., Mudrak E. (2020). Facilitators and Barriers to Preparing and Selling Whole Grain Snack Packs in New York City Corner Stores Participating in a Healthy Retail Program. J. Hunger Environ. Nutr..

[37] Thayer J.C., Morris V., Rollins J.V., Cook J. (2010). A Participatory Intervention to Address Adolescent Snack and Drink Purchasing Behaviors at Convenience Stores. J. Am. Diet. Assoc..

[38] NHANES Dietary Web Tutorial-Survey Orientation. http://medbox.iiab.me/modules/en-cdc/www.cdc.gov/nchs/tutorials/Dietary/Basic/PopulationMeanIntakes/intro.htm.

[39] Moshfegh A.J., Rhodes D.G., Baer D.J., Murayi T., Clemens J.C., Rumpler W.V., Paul D.R., Sebastian R.S., Kuczynski K.J., Ingwersen L.A. (2008). The US Department of Agriculture Automated Multiple-Pass Method reduces bias in the collection of energy intakes. Am. J. Clin. Nutr..

[40] (2020). NHANES Questionnaires, Datasets, and Related Documentation. Natl. Health Nutr. Exam. Surv..

